# Exploring the Potential of Personalized Dietary Advice for Health Improvement in Motivated Individuals With Premetabolic Syndrome: Pretest-Posttest Study

**DOI:** 10.2196/25043

**Published:** 2021-06-24

**Authors:** Sandra van der Haar, Femke P M Hoevenaars, Willem J van den Brink, Tim van den Broek, Mariëlle Timmer, André Boorsma, Esmée L Doets

**Affiliations:** 1 Wageningen Food & Biobased Research Wageningen University & Research Wageningen Netherlands; 2 Microbiology & Systems Biology Department TNO, Netherlands Organization for Applied Scientific Research Zeist Netherlands

**Keywords:** personalized nutrition, metabolic syndrome, dietary behavior, diet, metabolic, metabolic health, dietary advice, dietary feedback, digital health

## Abstract

**Background:**

Dietary quality plays an essential role in the prevention and management of metabolic syndrome (MetS).

**Objective:**

The aim of this pilot study is to organize personalized dietary advice in a real-life setting and to explore the effects on dietary intake, metabolic health, and perceived health.

**Methods:**

We followed a one-group pretest-posttest design and included 37 individuals at risk of MetS, who indicated motivation to change dietary behavior. For a period of 16 weeks, participants received personalized advice (t=0 and t=8) and feedback (t=0, t=4, t=8, t=12 and t=16) on dietary quality and metabolic health (ie, waist circumference, BMI, blood pressure, lipid profile, fasting glucose levels, and C-peptide). Personalized advice was generated in a two-stage process. In stage 1, an automated algorithm generated advice per food group, integrating data on individual dietary quality (Dutch Healthy Diet Index; total score 8-80) and metabolic health parameters. Stage 2 included a telephone consultation with a trained dietitian to define a personal dietary behavior change strategy and to discuss individual preferences. Dietary quality and metabolic health markers were assessed at t=0, t=8, and t=16. Self-perceived health was evaluated on 7-point Likert scales at t=0 and t=16.

**Results:**

At the end of the study period, dietary quality was significantly improved compared with the baseline (Dutch Healthy Diet Index +4.3; *P*<.001). In addition, lipid profile (triglycerides, *P=.*02; total cholesterol, *P*=.01; high-density lipoprotein, *P*<.001; and low-density lipoprotein, *P*<.001), BMI (*P*<.001), waist circumference (*P*=.01), and C-peptide (*P*=.01) were all significantly improved, whereas plasma glucose increased by 0.23 nmol/L (*P*=.04). In line with these results, self-perceived health scores were higher at t=16 weeks than at baseline (+0.67; *P*=.005).

**Conclusions:**

This exploratory study showed that personalized dietary advice resulted in positive effects on dietary behavior, metabolic health, and self-perceived health in motivated pre-MetS adults. The study was performed in a do-it-yourself setting, highlighting the potential of at-home health improvement through dietary changes.

**Trial Registration:**

ClinicalTrials.gov NCT04595669; https://clinicaltrials.gov/ct2/show/NCT04595669

## Introduction

### Background

Metabolic syndrome (MetS) is associated with a two-fold increased risk of cardiovascular diseases and a five-fold increased risk of type 2 diabetes [1,2]. Approximately 25% of adults globally are affected by MetS, and its prevalence increases with age [3,4]. MetS is defined by the coexistence of three or more of the following risk factors: abdominal obesity, high fasting blood glucose, reduced high-density lipoprotein (HDL) cholesterol, elevated fasting plasma triglycerides, and elevated blood pressure (BP) [5].

Unhealthy dietary habits are a major risk factor for developing MetS and are probably even more relevant than sedentary lifestyles [6]. Research has demonstrated dietary strategies that can be used to prevent or resolve MetS and associated metabolic abnormalities [6-9]. Adherence to a healthy diet rich in fruits, vegetables, whole grains, and oily fish, while limiting total fat, saturated fat, dietary cholesterol, added sugars, sodium, and excessive alcohol intake, has been shown to be effective in improving metabolic abnormalities associated with MetS [7,8,10]. Associations between dietary changes and individual risk factors for MetS have also been shown [11-21].

Personalized nutrition, that is, evidence-based dietary advice tailored toward an individual based on individual-specific information, is most likely an effective strategy to support dietary behavior change, resulting in measurable health benefits [22]. Previous studies have shown that personalized advice is more effective than giving a *one-size-fits-all* advice for improving dietary patterns, increasing physical activity and smoking cessation [23-30].

We distinguished two potential reasons for this effectiveness. First, each person receives advice that addresses the individual nutritional needs based on the person’s biology, thereby maximizing the individual health effect. In a study on healthy volunteers receiving placebo or anti-inflammatory dietary mix supplements, the inflammatory, oxidative, and metabolic responses were highly variable among individuals, suggesting different nutritional needs based on the person’s biology [31]. Indeed, the concept of personalized nutrition from a biological perspective began to emerge as extensively reviewed by van Ommen et al in 2017 [32].

A second reason for personalized nutrition being effective is increased adherence to the advice when it is made personal. Each person receives only the information based on their characteristics, rather than generic information based on the characteristics of the population. Therefore, people are more likely to pay attention and feel more involved, especially when the information is tailored to the personal level of motivation [23].

Celis-Moralis et al reviewed the evidence on personalized interventions and concluded that there is a strong need for further development, testing, and implementation of digitally delivered, evidence-based, personalized interventions that incorporate effective behavior change techniques (eg, personal goal setting and feedback on performance) and are delivered digitally [33,34]. In a web-based multicenter study, Forster et al [35] compared an automated feedback system with manual feedback and found good agreement between the manual and automated feedback systems, showing promise for the use of automated systems for personalizing dietary advice. With regard to scalability and expected contribution to sustained behavior change, new evidence on the effectiveness and acceptance of these digitally delivered interventions is highly relevant.

### Objectives

The primary aim of this pilot study is to organize personalized dietary advice in a real-life setting. We build upon the research described by Doets et al [36] by exploring the combined effects of dietary intake, metabolic health, and perceived health. As we aim to conduct real-life implementation, we are targeting individuals at risk of MetS, who are intrinsically motivated to change their dietary behavior to improve their health, as they are likely to be easy adopters of personalized advice.

## Methods

### Ethics Statement

All participants provided informed consent for inclusion before they participated in the study. The study was conducted in accordance with the Declaration of Helsinki, and the protocol was approved by the Ethics Committee of Tilburg University (file number NL61382.028.17).

### Recruitment and Screening

**Figure 1:**
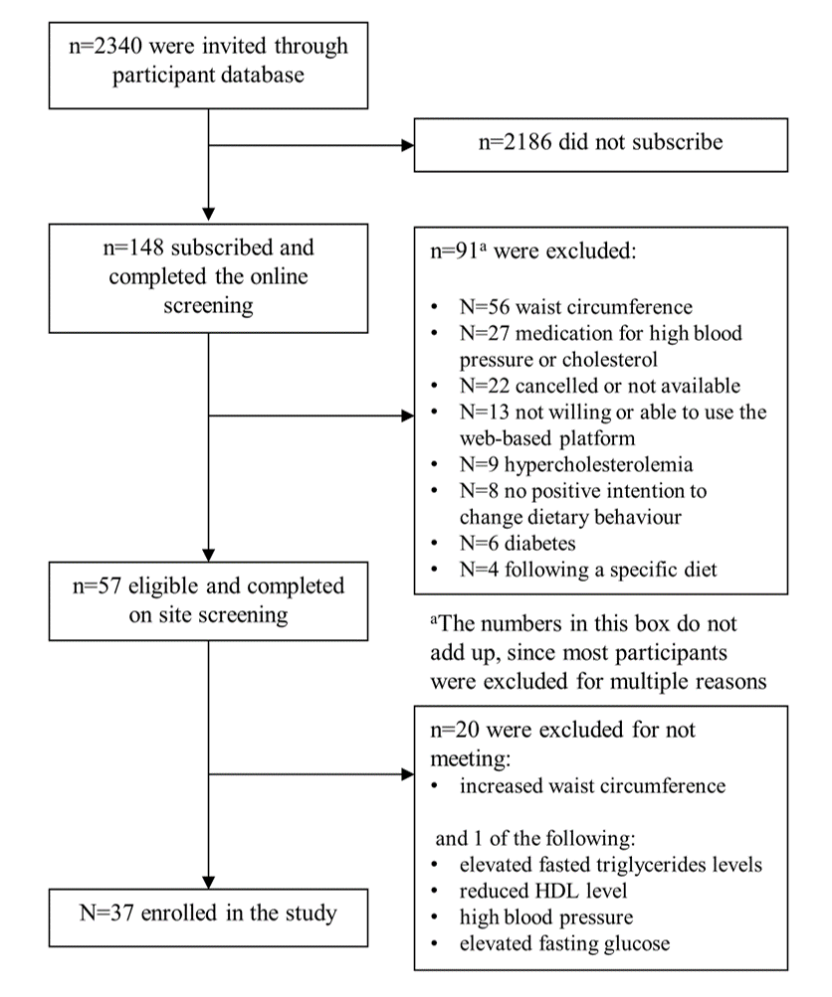
Flowchart of recruitment and screening procedure. HDL: high-density lipoprotein.

An overview of the recruitment procedure is shown in Figure 1. First, all members of the consumer database of Wageningen University & Research received an invitation letter for the study. Those interested in study participation completed a web-based screening questionnaire to verify the first set of inclusion and exclusion criteria: age ≥40 years, excessive waist circumference (self-reported ≥88 cm for women and ≥102 cm for men), positive intention toward changing dietary behavior, and willingness to use digital web-based applications during the study. To assess intention toward behavior change, we used an adapted version of 3 questions (7-point Likert scale) reported by Poinhos et al [37]. Participants with a mean score of ≥5 were considered to be motivated to change their dietary behavior. Exclusion criteria were as follows: taking medication known for its effects on blood glucose, cholesterol, or insulin; being diagnosed with diabetes or familial hypercholesterolemia; following a specific diet or having an alcohol consumption of >28 units (drinks) per week for men and >21 units per week for women.

To improve health and behavioral changes through personalized advice, we included individuals at risk of MetS (second set of inclusion criteria). Therefore, individuals eligible for study participation were invited for additional screening to verify whether they were at risk of MetS, defined as an excessive waist circumference (≥88 cm for women and ≥102 cm for men) combined with elevated fasted triglycerides levels (≥1.7 mmol/L), reduced HDL level (<1.03 mmol/L for men and <1.29 mmol/L for women), high BP (systolic: ≥130 mm Hg or diastolic: ≥85 mm Hg), or elevated fasting glucose (>5.6 mmol/L).

On the basis of the study by Doets et al [36], we performed a power calculation to estimate an adequate sample size to identify potential health effects. The effect size was based on the mean change in waist circumference (δ=1.85 cm, SD 2.67; significance level of 5%). The calculations revealed that a sample size of 16 would be sufficient to identify potential health effects based only on waist circumference. However, based on the sample sizes of previous pilot studies [36,38] and taking into account various combinations of MetS risk factors and potential dropout, we increased the number of participants to 40.

### Study Design

The study followed a one-group pretest-posttest design with a duration of 16 weeks (Table 1). Reviews of behavioral intervention studies have shown that a period of 16 weeks is minimal to allow the first stages of behavior change [39,40]. Our study targeted individuals who were motivated to change their dietary behavior; therefore, a period of 16 weeks was considered sufficient. As the aim of the study was to explore the potential effects of personalized nutrition in real life, no control group was included. The intervention consisted of personalized dietary advice in combination with feedback on dietary behavior and health status (ie, waist circumference, BP, cholesterol, glucose, BMI, C-peptide, and triglycerides) at set time points throughout the study.

**Table 1:** Overview of study design: measurements, interventions, and planning.

Measurements and characteristics of the intervention			Timepoints (weeks), t
**Diet**			
	Diet quality (Dutch Healthy Diet Index) per food category and total score	0, 8, 16	
	Carotenoids in blood (biomarker fruit and vegetable intake)	0, 8, 16	
	Food purchase data at retailer (via customer card)	4, 12	
**Subjective health**			
	Self-perceived health questionnaire	0, 16	
**Consumer experiences**			
	Evaluation questionnaire	16	
**Metabolic health**			
	Waist circumference	0, 8, 16	
	BMI	0, 8, 16	
	Blood pressure	0, 8, 16	
	Lipid profile (total cholesterol, HDL^a^, LDL^b^, and triglycerides)	0, 8, 16	
	Fasting glucose	0, 8, 16	
	C-peptide	0, 8, 16	
**Personalized advice**			
	Stage 1: automated advice based on individual diet quality and metabolic health status	0, 8	
	Stage 2: telephone consultation with dietitian to define behavioral change strategy and discuss personal preferences	0, 8	
**Feedback**			
	Diet quality discussed in telephone consultations with dietitian	0, 8	
	Alternatives for product purchases in email messages from dietitian	4, 12	
	Metabolic health via web-based platform	0, 8, 16	
	Integrated personal health score via web-based platform	0, 16	

^a^HDL: high-density lipoprotein.

^b^LDL: low-density lipoprotein.

### Study Procedures

During the 3 test days (t=0, t=8, and t=16 weeks), participants arrived in the morning in a fasted state to Wageningen University and Research, the Netherlands. Metabolic health parameters were assessed by trained research nurses using do-it-yourself devices, following standard operating procedures. Total cholesterol, HDL, low-density lipoprotein (LDL), and triglycerides were measured in finger-prick blood using the Mission Cholesterol 3-in-1 device (Acon Labs Inc). Glucose levels were assessed using a MediTouch 2 blood glucose meter (Medisana). BP was measured using a Medisana upper arm BP monitor. Both glucose and BP were measured twice for each participant, and the average result was used as input for feedback and personalized advice.

### Dietary Quality

Dietary quality was assessed by using a web-based version of the Dutch Healthy Diet Index (DHDI; *Eetscore*, Division of Human Nutrition, Wageningen University) [41]. The DHDI evaluates adherence to the Dutch dietary guidelines per food category (score 1-10) and total score (score 8-80). In this study, we focused on food categories that have been shown to be effective for improving metabolic abnormalities due to reducing calorie intake or through direct effects on metabolic parameters. These food categories are fruit and vegetables [16,17,19], wholegrain products [13,14,16], dairy products [17,18,20], fish [10,16,21], saturated fat (butter, meat, and snacks) [12], and sugar-containing beverages [17]. The DHDI results were used as input for the dietary advice tool.

### Metabolic Health Parameters

Waist circumference was determined directly over the skin at the midpoint between the lower part of the last rib and the top of the hip. Body weight was recorded on a calibrated weighing scale to the nearest 0.1 kg. Finger-prick blood was blotted on dried blood spot (DBS) cards (Protein Saver TM 903R Cards, Whatman). To suppress the degradation of carotenoids in the DBS samples, the first two circles in the DBS cards were impregnated with a proprietary stabilizing solution supplied by Vitas AS. After air drying for several hours, the cards were stored in airtight resealable aluminum bags (Whatman) with a desiccant pouch (Reàl Marine A/S Stavanger) to remove any moisture from the DBS cards. C-peptide and carotenoids were assessed via high-performance liquid chromatography with UV detection and liquid chromatography-mass spectrometry (Vitas AS) [42]. In brief, from each DBS, 3·2-mm disks were punched out and mixed with distilled water. Next, proteins were precipitated and carotenoids were extracted with isopropanol containing an internal standard (β-Apo-8 carotenal, Sigma-Aldrich). An aliquot of the isopropanol phase was analyzed using a 1100-series HPLC-UV system with a 1260 diode array detector (453 nm; Agilent Technologies). Separation was performed on a 3-mm YMC C30 column (150 mm×4·6 mm internal diameter, YMC).

### Personalized Dietary Advice

At t=0 and t=8 weeks, the participants received personalized dietary advice. Personalized advice was generated in a two-stage process. During stage 1, the content of the advice was defined based on individual dietary habits (ie, DHDI and carotenoid levels as a biomarker of fruit and vegetable intake) and parameters of metabolic health. The results of these measurements were added to an automated personalized dietary advice system. First, the algorithm evaluated per food category (dairy, fats and oils, fish, fruit, nuts, sugar-containing beverages, vegetables, and wholegrain products) whether intake and nutrient status were sufficient based on predefined cut-off levels. If intake or nutrient status was insufficient, the food category was included in the advice. Second, the system evaluated the presence of metabolic abnormalities. If metabolic abnormalities were present, relevant food categories were included in the advice to emphasize the importance of adequate intake for a specific food category.

Stage 2 included a telephone consultation of 45-60 minutes, during which a trained dietitian discussed the system-generated advice with the participant following a standard protocol. During the consultation, a personal dietary behavior change strategy was defined by adapting the advice from stage 1 to individual preferences (eg, number of food groups to work on, selection of alternative products, and adjustment of portion sizes). In Table S1 of Multimedia Appendix 1, the steps followed by the dietitian are displayed. A summary of the dietary behavior change strategy was available to the participants through a web-based personal study portal. The provided dietary advice was in line with the national dietary recommendations provided by the Health Council of the Netherlands and the Netherlands Nutrition Centre [43,44].

### Feedback

Feedback on behavioral parameters was provided to participants by a dietitian as part of the individual telephone consultations at t=0 and t=8 weeks and via email at t=4 and t=12 weeks. The feedback by telephone addressed adherence to Dutch dietary guidelines based on the DHDI. The feedback by email addressed healthy alternatives for recent product choices and was based on purchase data registered on a supermarket customer card that participants were asked to share with the research team. Feedback on metabolic health parameters (ie, waist circumference, BMI, BP, glucose, cholesterol, C-peptide, and triglycerides) was directly communicated to the subjects via a web-based personal study portal at t=0, t=8, and t=16 weeks.

Furthermore, at t=0 and t=16 weeks, each participant received an integrated personal health score based on their metabolic health parameters.

### Personal Health Score

The personal health score was produced using a so-called *health space* model that was created on basis of the principle of van den Broek et al [45]. This type of model can produce an individual unitless score based on personal data that correspond to the individuals’ health status after being trained on the data of two reference groups. The model in this study was trained on two reference groups from an independent data set (National Health and Nutrition Examination Survey 2003-2004 [2003]; [46]), a group of healthy subjects with no diagnostic characteristic for MetS versus subjects diagnosed with MetS (Table 2). The MetS group was selected based on the MetS definition of the International Diabetes Federation [47]. In turn, 135 subjects in the healthy group were selected from all available subjects by constraining BMI between 18 and 25 kg/m^2^. Of these 135 subjects, the top 10 were selected based on their aggregated rank.

**Table 2:** Demographics and metabolic health parameters of study participants and the health space reference groups (N=85).

Variable			Study participants (n=34)		Healthy reference^a^ (n=10)		MetS^b^ reference^a^ (n=41)
**Sex, n** **(%)**							
	Male	9 (26)		5 (50)		19 (46)	
	Female	25 (74)		5 (50)		22 (54)	
Age (years), mean (SD)			61 (8.2)		57.6 (16.2)		54 (21.0)
BMI (kg/m^2^), mean (SD)			29.9 (4.18)		21.3 (1.88)		31.1 (5.68)
Waist circumference (cm), mean (SD)			102 (11.4)		83.2 (4.68)		105 (10.8)
Total cholesterol (mmol/L), mean (SD)			6.23 (0.78)		5.32 (1.10)		4.77 (1.02)
HDL^c^ cholesterol (mmol/L), mean (SD)			1.14 (0.27)		1.49 (0.37)		1.01 (0.13)
LDL^d^ cholesterol (mmol/L), mean (SD)			4.34 (0.74)		3.12 (1.02)		2.73 (0.99)
Triglycerides (mmol/L), mean (SD)			1.67 (0.85)		1.57 (0.52)		2.25 (0.95)
Glucose (mmol/L), mean (SD)			5.61 (0.65)		5.33 (0.70)		7.07 (3.08)
C-peptide (nmol/L), mean (SD)			0.52 (0.33)		0.75 (0.40)		1.46 (0.90)
Systolic blood pressure (mm Hg), mean (SD)			135 (18.0)		138 (18.5)		128 (21.5)
Diastolic blood pressure (mm Hg), mean (SD)			78.6 (9.54)		77.7 (14.0)		67.4 (18.6)

^a^Data for the reference groups were obtained from the National Health and Nutrition Examination Survey 2003-2004 (CDC 2003) [46].

^b^MetS: metabolic syndrome.

^c^HDL: high-density lipoprotein.

^d^LDL: low-density lipoprotein.

This aggregated rank is based on the features included in the trained model, where the highest rank corresponds to the healthiest values of these features. The aggregated rank of this collection of features was calculated using the *robust rank aggregation* algorithm proposed by Kolde et al [48]. The data used in the training of this model were taken from the National Health and Nutrition Examination Survey 2003-2004 data set (CDC 2003) [46].

A multivariate mixed-effects regression model was subsequently fitted to the data from the two selected reference groups with good classification performance with an accuracy of 99% and a Cohen κ coefficient of 0.94. The model includes triglycerides, LDL cholesterol, HDL cholesterol, glucose, and C-peptide as fixed effects and sex as a random effect. The random effect was included to allow for sex differences in the final model coefficients. Table S2 in Multimedia Appendix 1 shows the standardized contributions of each feature in the final model. Finally, individual health scores were calculated by feeding participants’ metabolic health data into the regression model.

### Self-Perceived Health and Consumer Experiences

At baseline and at the end of the study, participants reported self-perceived health, self-perceived healthiness of the diet, and satisfaction with the diet using a 7-point Likert scale ranging from 1=very unhealthy to 7=very healthy and 1=very unsatisfied to 7=very satisfied.

At the end of the study, participants filled out an evaluation questionnaire on personal experiences regarding advice, feedback, and the digital platform (statements on 7-point Likert scales, ranging from 1=completely disagree to 7=completely agree).

### Statistical Analyses

Data on DHDI scores and metabolic health were analyzed using linear mixed models with *time* (t=0 vs t=8 vs t=16 weeks) as a fixed effect and *subject* as a random effect. Self-perceived health data were evaluated using ordinal mixed regression models with the same model structure. Post hoc analyses were performed on these models to identify significant differences between the individual time points. In the linear mixed model, observations with an absolute residual >3 times the root mean square error of the model were treated as statistical outliers.

In addition, Pearson correlation coefficients were calculated between the Δ of the single dietary behavior variables and the single metabolic health variables and between the Δ of dietary behavior and metabolic health variables. Only significant correlations that could be visually confirmed in the scatterplots were regarded as reliable (Figure S1 in Multimedia Appendix 1).

For the analyses of the individual food categories, only participants that actually incorporated the specific food category in their dietary behavior change strategy were included. *P* values reported from the mixed model post hoc tests were adjusted for multiple comparisons following the Benjamini-Hochberg procedure [49].

Statistical significance was set at *P*<.05 for all analyses. Statistical analyses were performed using R version 3.4.3 (R Core Team).

## Results

### Baseline Characteristics

A total of 37 individuals were enrolled in this study. During the study period, 3 subjects dropped out: 1 participant no longer met the inclusion criteria, and the other 2 experienced too many difficulties in using the web-based platform. The baseline characteristics of the 34 participants who completed the intervention as well as the reference populations used for modeling the health score are summarized in Table 2. Next to excessive waist circumference, most participants presented multiple risk factors for MetS. High BP, high glucose, high triglycerides, and low HDL were present in 21, 16, 14, and 22 subjects, respectively.

### Effect of Personalized Advice on Dietary Quality

Most subjects (33/34, 97%) were provided with advice on multiple food categories in their individual dietary behavior change strategies. One participant chose to focus on only one food category. Advice was provided most frequently on vegetables (31/34, 91%), followed by oils and fat (21/34, 62%), nuts (20/34, 59%), wholegrain products (19/34, 56%), dairy (14/34, 41%), fish (12/34, 35%), fruit (9/34, 27%), and sugar-containing beverages (3/34, 9%). The mean DHDI scores over time per food category are shown in Table 3. An improvement over time was observed for wholegrain products (+1.6; *P*=.009; 19/34, 56%), nuts (+2.2; *P*=.009; 20/34, 59%), and total DHDI (+4.3; *P*<.001; 34/34, 100%). The change in total DHDI was significantly correlated in decreasing order with the change in oils and fats score (ρ=0.62; *P*<.001), nuts score (ρ=0.62; *P*<.001), dairy score (ρ=0.55; *P*<.001), fish score (ρ=0.39; *P*=.03), and fruit score (ρ=0.39; *P*=.02).

**Table 3:** Dutch Healthy Diet Index per food category (score 1-10) and total score (score 8-80) and total carotenoids (µmol/L) at baseline, 8 weeks, and 16 weeks.

Food category^a^	DHDI^b^ score, mean (SD)				*P* value
	t=0 weeks	t=8 weeks	t=16 weeks		
Vegetable intake (n=31)	6.6 (2.9)	7.5 (3.0)	7.1 (3.3)	.53	
Fruit intake (n=9)	5.8 (3.3)	8.2 (2.0)	8.4 (2.0)	.70	
Intake of oils and fats (n=21)	3.7 (3.9)	3.6 (4.1)	3.5 (3.8)	.85	
Fish intake (n=12)	6.6 (3.2)	7.5 (2.6)	8.6 (1.9)	.18	
Intake of wholegrain products (n=19)	6.3 (2.5)^c^	7.7 (2.8)^c^	7.9 (2.8)^c^	.009	
Dairy intake (n=14)	3.1 (2.6)	3.8 (3.1)	4.1 (2.7)	.84	
Nut intake (n=20)	6.2 (3.3)^c^	7.0 (2.6)^c^	8.4 (2.5)^c^	.009	
Intake of sugar-containing beverages (n=3)	1.9 (1.8)	5.5 (5.0)	6.6 (2.8)	—^d^	
Total DHDI (sum of all food categories; n=34)	52.9 (13.1)^c^	56.5 (11.3)^c^	57.2 (11.5)^c^	<.001	
Carotenoid levels in blood (µmol/L; t=0, n=36; t=8, n=34; t=16, n=33)	1.21 (0.43)	1.39 (0.46)	1.42 (0.56)	.66	

^a^Only participants who included the specific food category in their individual dietary behavior change strategy are included in the analysis.

^b^DHDI: Dutch Healthy Diet Index.

^c^No significant difference following the post hoc analysis.

^d^Not available (as the sample size was not sufficient to obtain reliable statistical output).

### Effect of Personalized Advice on Metabolic Health Parameters and Health Score

After 16 weeks of intervention, triglycerides, HDL cholesterol, LDL cholesterol, BMI, waist circumference, C-peptide, and HOMA-IR were all significantly improved (Table 4). Plasma glucose increased significantly by 0.23 nmol/L (*P*=.04; Table 4). The overall health score significantly improved by 0.27 points on a scale from 1 (MetS reference) to 2 (healthy reference; *P*<.001; Table 4). Improvement in HDL cholesterol had the strongest overall impact on the increase in the health score, indicated by the high correlation between their changes from week 0 to week 16 (ρ=0.97; *P*<.001), whereas glucose, C-peptide, triglycerides, and LDL cholesterol changes were not significantly correlated with the increase in health score.

**Figure 2:**
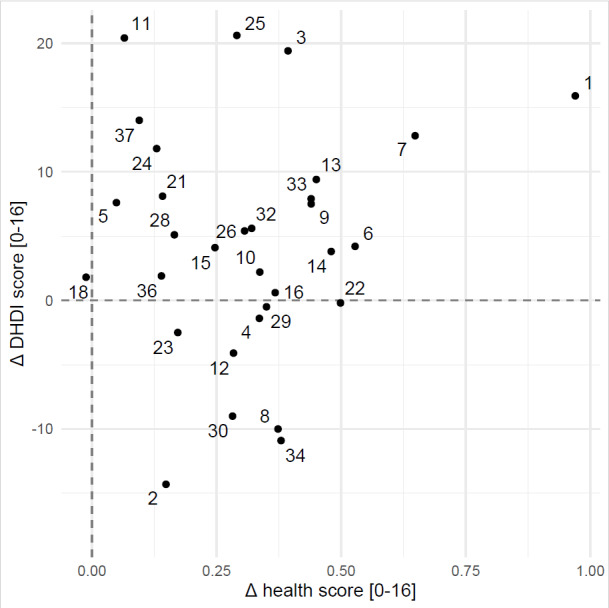
Association between the Δ dietary scores and Δ health scores calculated between week 0 and week 16 of the study.

No significant correlation was found between the changes in the total DHDI and health scores (ρ=0.12; *P*=.52; Figure 2). In particular, 9 subjects who did not improve total DHDI still showed an increased health score after 16 weeks (Figure 2). Significant correlations were found between changes in the total dietary scores and triglycerides (ρ=0.58; *P*<.001).

**Table 4:** Metabolic health parameters assessed at t=0, 8, and 16 weeks.

Parameter	t=0 weeks, mean (SD)	t=8 weeks, mean (SD)	t=16 weeks, mean (SD)	*P* value
Glucose (mmol/L)	5.61 (0.67)^a^	5.63 (0.64)^a^	5.84 (0.63)^a^	.04
C-peptide (nmol/L)	0.52 (0.32)^a^	0.52 (0.23)^a^	0.43 (0.16)^a^	.01
HOMA-IR^b^	7.45 (5.27)^a^	7.42 (3.95)^a^	6.31 (2.57)^a^	.049
Triglycerides (mmol/L)	1.67 (0.86)^a^	1.43 (0.60)^a^	1.39 (0.55)^a^	.02
Total cholesterol (mmol/L)	6.23 (0.78)	5.91 (0.84)^a^	5.90 (0.86)^a^	0.01
HDL^c^ cholesterol (mmol/L)	1.14 (0.28)^a^	1.09 (0.28)^a^	1.44 (0.36)^a^	<.001
LDL^d^ cholesterol (mmol/L)	4.34 (0.74)^a^	4.18 (0.79)^a^	3.87 (0.78)^a^	<.001
Systolic blood pressure (mm Hg)	135 (18.2)	133 (13.7)	132 (17.1)	.70
Diastolic blood pressure (mm Hg)	78.6 (9.60)	80.3 (8.93)	79.6 (9.77)	.30
BMI (kg/m^2^)	29.9 (3.94)^a^	29.4 (3.60)^a^	29.2 (3.66)^a^	<.001
Waist circumference (cm)	102 (11.5)^a^	100 (9.43)^a^	99.4 (8.86)^a^	.01
Health score (arbitrary units)	1.30 (0.31)	1.23 (0.30)	1.57 (0.32)	<.001

^a^No significant difference following the post hoc analysis.

^b^HOMA-IR: homeostatic model assessment–insulin resistance; calculated based on glucose and C-peptide [50].

^c^HDL: high-density lipoprotein.

^d^LDL: low-density lipoprotein.

### Self-Perceived Health and Consumer Experiences

The mean scores for self-perceived health, self-perceived healthiness of the diet, and satisfaction with the diet, as reported by the participants at baseline and end, are shown in Table 5. All three scores were significantly improved at the end of the study compared with baseline (self-perceived health: +0.67, *P*=.005; self-perceived healthiness of the diet: +1.06, *P*<.001; satisfaction with the diet: +0.94, *P*=.001). In addition, participants reported positive mean scores on how helpful the personalized advice and feedback were to improve their diet. In addition, they were positive about continuing the advice after completion of the study and on advising other people to obtain personalized advice, as in this study. Participants would not be willing to pay for (parts of) this program.

**Table 5:** Self-perceived health, self-perceived healthiness of the diet, and satisfaction with the diet as reported at t=0 and 16 weeks.

Self-perceived health items			t=0 weeks, mean (SD)		t=16 weeks, mean (SD)		*P* value
Self-perceived health^a^			4.68 (1.07)		5.35 (1.10)		.005
Self-perceived healthiness of diet^a^			4.50 (1.05)		5.56 (0.96)		<.001
Satisfaction with diet^b^			4.35 (1.39)		5.29 (1.14)		.001
**Consumer experiences^c^**							
	The personalized advice helped me to improve my diet	—^d^		5.7 (1.5)		—	
	The feedback helped me to improve my diet	—		5.4 (1.4)		—	
	If possible I would continue taking part in this program	—		4.7 (2.0)		—	
	I would recommend people in my surroundings to obtain personalized advice like in this study	—		5.0 (1.8)		—	
	I would be willing to pay for this program	—		2.5 (1.7)		—	

^a^7-point Likert scale, ranging from “very unhealthy” to “very healthy.”

^b^7-point Likert scale, ranging from “very unsatisfied” to “very satisfied.”

^c^7-point Likert scale, ranging from “completely disagree” to “completely agree.”

^d^Consumer experiences were only assessed at the end of the study (t=16).

## Discussion

### Principal Findings

With the aim of exploring the combined behavioral and metabolic health effects in relation to personalized nutrition, we have shown that personalized dietary advice delivered through an automated advice system and discussed by a dietitian with the participant has a significant positive effect on dietary behavior, with a concurring beneficial impact on metabolic health in consumers at risk of MetS. Moreover, the perception of health and healthiness of and satisfaction with the diet improved.

Most earlier studies have shown the impact of personalized dietary advice either on dietary intake or on metabolic health parameters. In this study, we build upon the research described by Doets et al [36] by focusing on the combined analysis of dietary intake, metabolic health, and perceived health. In this previous study, we evaluated the potential of digitally delivered personalized lifestyle advice for improving well-being compared with general dietary advice in a population of active seniors. Well-being was operationalized by self-perceived health and well-being as well as biological measures, including markers of metabolic health and physical function tests. Despite some clear limitations with respect to the target population (eg, already having a healthy diet), the short intervention duration (9 weeks), the provided feedback (all participants, including the control group, received individual feedback on their health and well-being), and the intake tools used for monitoring dietary behavior, the results showed that personalized lifestyle advice might have the potential to improve health outcomes as compared with general lifestyle advice.

Compared with Doets et al [36], the study design was improved by prolonging the study duration, including an individual behavior change strategy, selecting an at-risk population, and increasing the frequency of feedback on individual metabolic health.

Interestingly, our results revealed no correlation between the effect on dietary behavior and metabolic health, although both variables showed a significant improvement.

In our study, there was a large variation in the personalized advice between participants, as the advice was tailored to individual metabolic health status as well as dietary quality. Most participants in our study sample incorporated improved intake of vegetables, oils and fats, nuts, and wholegrain products in their behavior change strategy (n≥19). Among these, participants seemed to comply with the advice for wholegrain products and nuts, especially as these two food groups significantly improved over time. In contrast, no changes were observed in vegetables, oils, and fats. These results suggest that it is easier for motivated participants to replace refined products with wholegrain products or to include nuts in their dietary patterns as compared with increasing vegetable intake or changing the type of fat for the preparation of meals or for bread spread. Previous systematic reviews have shown significant pooled effects of dietary advice interventions on increased intake of fruits, vegetables, total fiber, and total fat [51-53]. However, most of these reviewed studies focused on changing a single dietary behavior aspect in line with general recommendations rather than optimizing dietary intake in view of improved individual health. A recent study by Rijnaarts et al [54] showed that providing fiber-rich alternatives via an automated, personalized advice system increased adherence to recommendations as compared with generic advice, confirming the effectiveness of a personalized advice system and replacing refined products with wholegrain products.

Although our results indicated an improvement over time for fruit, fish, dairy, and intake of sugar-containing beverages, these effects turned out to be nonsignificant as only a small number of individuals included these food groups in their behavior change strategy (n≤14).

Several studies have reviewed available behavior change techniques that are effective for dietary behavior change [55-57]. They demonstrated that tailoring, instructions, goal setting, and feedback are effective intervention elements for evoking dietary behavior change. The personalized intervention we used in the study combined several behavior change techniques to facilitate behavior change: feedback on health (what is the actual health situation), feedback on behavior (which dietary changes are relevant for the individual based on parameters of health and diet), advice on how to change behavior (how these changes can be made in terms of product choice), and individual goal setting (what does the individual want to change, ie, behavior change strategy) [57]. Problem solving and social comparison seem to be other relevant behavior strategies to further improve our intervention. These strategies may be especially relevant for improving compliance with advice on fruit and vegetable intake [52,55].

### Effectiveness of Intervention on Metabolic Health

We hypothesized that by optimizing the quality of the diet in terms of adherence to Dutch dietary guidelines on specific food groups, we were able to improve the metabolic health of our participants. Our analysis indeed showed significant improvements in metabolic health; however, whether these effects were due to improvements in diet quality could not be substantiated. Previous reviews have shown that the restriction of total energy intake, carbohydrate, or fat is a successful strategy to improve metabolic health status. Furthermore, enriching the diet with monounsaturated fatty acids (nuts and olive oil) or omega-3 fatty acids (fish) has been proven effective, especially in improving lipid profiles [7,58,59].

Although the absolute health score is also determined by subtle changes in triglycerides, glucose, C-peptide, and LDL cholesterol, it seems that the relatively strong Δ HDL cholesterol is the main driver for the change in the health score. It is known from the literature that HDL levels are affected by the increased consumption of fish and unsaturated fatty acids and decreased consumption of saturated fatty acids [60,61]. Interestingly, in our data, the increase in HDL cholesterol could not be significantly related to any specific dietary improvement. Apart from the fact that the statistical power may not have allowed it, this observation may be related to a confounding effect of activity. Results from a meta-analysis showed a highly significant relationship between physical activity and HDL cholesterol levels [62].

### Effectiveness of Intervention on Self-Perceived Health

Self-perceived health summarizes the objective and subjective aspects of health within the perceptual framework of an individual. Some studies suggest that although the criteria for judging health status may vary between individuals, it is a valid indicator of overall health status and use of health services [63,64]. However, the cross-sectional association between actual metabolic health and perceived health remains unclear [65]. Previous intervention studies have shown a clear link between improved health behaviors and better self-perceived health scores, supporting our findings [64,66,67]. From the perspective of maintaining behavior change, improvement in self-perceived health in the short term is highly relevant as it helps individuals to stay motivated, allowing the behavior change to persist over a longer period.

### Lessons Learned

Participants may have become more aware of their dietary behavior throughout the study, which may have influenced their answers to the DHDI questionnaire, causing a learning bias [68]. Together with the fact that no control period was included, this may have influenced the dietary scores over time. In future studies, it is recommended to include a learning period before the start of the study to minimize the effect of learning.

No control group was included, which is a general challenge in studies investigating the efficacy of personalized nutrition. Therefore, it is not possible to separate the effect of diet from the potential effect of general health improvement as a behavioral consequence of taking part in the study. A semiplacebo control may be reached by comparing personalized advice with generic advice [54,69] or by allowing participants to be their own control by starting with a free-living run-in period without intervention. Furthermore, N-of-1 (or single-subject) study designs focusing on one individual could be a good fit to study research questions related to personalized dietary advice in the future. In N-of-1 designs, the optimal intervention for a specific individual is studied rather than an average individual from a target population.

Although we could confirm the assumption that personalized dietary advice is effective in improving both overall dietary behavior (total DHDI score) and overall metabolic health (health score), interestingly, there was no significant correlation. It should be noted that the pilot study only included 34 individuals, all of whom received personalized dietary advice. In addition, there are some limitations to the DHDI score, in which each food category is weighted equally in the total score. An adjusted total score in which the food categories relevant for MetS would outweigh the other food groups could possibly reveal a significant effect.

In addition to the positive effects of improved dietary quality, previous research has also demonstrated the beneficial effects of moderate- to high-intensity physical activity training on lipid profile, BP, and C-reactive protein [70,71]. In our study, we did not provide any advice on increasing physical activity; however, the study participants were invited to use a health watch, providing general feedback on daily activity levels. Owing to unforeseen practical reasons, these health watches were only available during the second half of the study period. Therefore, we were not able to evaluate possible changes in activity levels during the study. For future studies, it is highly recommended to include physical activity monitoring using either a device or a validated questionnaire.

Contrary to our expectations, these data illustrate that positive effects at the population level are not necessarily indicative of associations between diet and health. We can thus conclude that personalized dietary advice works for dietary behavior and health, but the data did not allow us to conclude that metabolic health was improved as a consequence of dietary improvement. A larger sample size with a more equal distribution of men and women and the addition of a control group to the study design are warranted to further investigate and understand the association between diet and health at the individual level. Furthermore, a follow-up after a longer period (eg, 6 months or 1 year) would allow to determine whether initiated behavior changes are maintained over time.

### Conclusions

In this exploratory pilot study in individuals at risk for MetS and motivated to change behavior, personalized dietary advice was indicative of positive effects on self-perceived health, dietary behavior, and metabolic health. The lack of association between diet and health improvement is reflective of the individual nature of diet-health relations and underlines the need for an integrated analysis focusing on individual improvements. The study was performed in a do-it-yourself setting, highlighting the potential of evidence-based at-home improvement of health through dietary changes. Follow-up studies are needed to confirm these effects and evaluate the maintenance of dietary behavioral changes.

## Appendix

Multimedia Appendix 1 An overview of the telephone consultation in stage 2 of the personalized advice with the trained dietitian (example of one food group for one participant) and standardized coefficients of the features in the health space model.

## Abbreviations

BP: blood pressure
DBS: dried blood spot
DHDI: Dutch Healthy Diet Index
HDL: high-density lipoprotein
LDL: low-density lipoprotein
MetS: metabolic syndrome
