# Supplementary material for: Exploring the Potential of Personalized Dietary Advice for Health Improvement in Motivated Individuals With Premetabolic Syndrome: Pretest-Posttest Study
Source: JMIR Form Res. 2021 Jun 24;5(6):e25043. doi: 10.2196/25043 (PMC8277310; doi:10.2196/25043)
Supplement: Multimedia Appendix 1 [file formative_v5i6e25043_app1.docx]

**Table S1**. An overview of the telephone consultation in stage 2 of the personalized advice with the trained dietitian (example of one food group for one participant).

| Items in the consultation | Sub-items |
| --- | --- |
| Step 1: Dietary habits and preferences | 1. Experience with weight loss diets |
|  | 1. Food allergies or intolerances |
|  | 1. Dietary habits and eating moments |
|  | 1. Other dietary preferences or dislikes |
| Step 2: Behavior change strategy | 1. Discuss automatically generated advice by the algorithm |
|  | 1. Discuss which food groups to focus on (one or multiple) |
|  | 1. Current dietary habits regarding this food group^a^ |
|  | 1. Assess willingness to change consumption behavior |
|  | 1. Define strategy:  - Increase portion size or number of consumption moments - Replacements within food group - Replacements from other food group(s) |
|  | 1. Provide advice and tips in line with strategy |
| Step 3: Summary and closure | 1. Summary of final advice for all food groups |
|  | 1. Record of the advice put on personal digital platform |

^a^ Steps 2 c-f were repeated for each food group as decided to focus on in step 2b

**Table S2.** Standardized coefficients of the features in the health space model.

| Feature | Coefficient |
| --- | --- |
| Triglycerides | +0.01 |
| LDL cholesterol | -0.12 |
| HDL cholesterol | +0.19 |
| Glucose | -0.18 |
| C-peptide | -0.27 |
| LDL cholesterol x HDL cholesterol | 0.14 |
| Glucose x C-peptide | 0.30 |
